# A New Residual Dense Network for Dance Action Recognition From Heterogeneous View Perception

**DOI:** 10.3389/fnbot.2021.698779

**Published:** 2021-06-22

**Authors:** Xue Yang, Yin Lyu, Yang Sun, Chen Zhang

**Affiliations:** ^1^College of Music, Huaiyin Normal University, Huai'an, China; ^2^College of Software, Shenyang Normal University, Shenyang, China; ^3^College of Sports Art, Harbin Sport University, Harbin, China

**Keywords:** dance action recognition, residual model, dense connection network, batch normalization, ELU

## Abstract

At present, part of people's body is in the state of sub-health, and more people pay attention to physical exercise. Dance is a relatively simple and popular activity, it has been widely concerned. The traditional action recognition method is easily affected by the action speed, illumination, occlusion and complex background, which leads to the poor robustness of the recognition results. In order to solve the above problems, an improved residual dense neural network method is used to study the automatic recognition of dance action images. Firstly, based on the residual model, the features of dance action are extracted by using the convolution layer and pooling layer. Then, the exponential linear element (ELU) activation function, batch normalization (BN) and Dropout technology are used to improve and optimize the model to mitigate the gradient disappearance, prevent over-fitting, accelerate convergence and enhance the model generalization ability. Finally, the dense connection network (DenseNet) is introduced to make the extracted dance action features more rich and effective. Comparison experiments are carried out on two public databases and one self-built database. The results show that the recognition rate of the proposed method on three databases are 99.98, 97.95, and 0.97.96%, respectively. It can be seen that this new method can effectively improve the performance of dance action recognition.

## Introduction

In recent years, with the rapid development of sensor technology in intelligent devices, many application fields such as human daily behavior recognition, health detection and health care guidance are rising rapidly (Li et al., 2018a; Yu et al., 2020). Early identification of human daily behavior is mainly to collect user behavior information by binding special sensors on fixed parts of human body. At present, with the popularity and rapid development of intelligent devices, the sensor information can be directly obtained when you carry it with you, and the information can be used to identify user behavior anytime and anywhere without affecting the normal work, study and life of users. However, due to the various brands and types of smart devices, the different locations carried by users and the differences in the performance of built-in sensors and other factors, the difficulty of human behavior recognition based on sensor information is increased, which affects the accuracy of recognition (Jisi and Yin, 2021).

In order to improve the accuracy of human daily behavior recognition, researchers have made some achievements in human behavior recognition based on the acquisition of sensing data from mobile devices. It is mainly reflected in three aspects:

1) Feature optimization is conducted through the data collected by the acceleration sensor. For example, an unsupervised behavior feature extraction method combining dense optical flow trajectory and sparse coding framework was proposed (Xiaojian and Xiaoqin, 2016). The recognition accuracy of jogging, fast running and walking was up to 85%. Chen et al. (2011) proposed a behavior recognition feature optimization method based on ant colony algorithm. The accuracy of behavior recognition reached to 89%, and the time complexity was also relatively decreased. Tanişik et al. (2013) proposed a behavior recognition method based on implicit conditional random field, which was characterized by the contour of human action sequence images, and the average recognition accuracy reached to 91.4%.
2) The behavior recognition model is reconstructed by proposing a new algorithm or an improved algorithm. For example, a hybrid expert model based on intelligent devices was proposed to recognize human behavior (Lee and Cho, 2014), the highest recognition accuracy was 92.56%. Thiemjarus (2010) proposed a daily behavior localization model based on the change of reference coordinates with the accuracy of 90.42%. Morillo et al. (2015) proposed an adaptive behavior recognition method based on multi-sensor data fusion using the coupled hidden Markov model, and the recognition accuracy of standing, walking, sitting, and lying actions exceeded 84%.
3) The multi-classifier system theory in ensemble learning is used to build the model, in which the fusion algorithm has become the core of many researchers. Zhang and Zhang (2011) proposed a selective ensemble learning algorithm to improve the prediction and classification efficiency of the ensemble learning machine and reduce its storage requirements. Kalid et al. (2020), the generalized combination rule of multi-classifier system based on genetic algorithm for parameter estimation was adopted to carry out pattern classification. Heng et al. (2016), the output of multiple extreme speed learning machines was used for simple mean algorithm fusion processing, and the recognition accuracy of the final model output was 3.6% higher than that of a single extreme speed learning machine. However, these methods the high-level semantic information is ignored in the fusion process.

Although the traditional recognition methods have achieved good results, the recognition process is complex and often requires manual intervention. The image features extracted by manually design are usually the shallow features of the image with limited expression ability and insufficient effective feature information. Moreover, the robustness of manually design method is poor and it is greatly affected by external conditions. With the development of deep learning and the improvement of hardware environment, the deep learning methods for action image recognition have become the focus of research. Deep learning convolutional neural network has a strong feature expression ability and does not need to manually design features. It has achieved good results in the fields of image classification, image segmentation and target detection (Yang et al., 2020). However, due to the strong learning ability of the network model, the non-linear relationship between input and output is complex, and the phenomenon of over-fitting is easy to appear. Moreover, the training of the network needs a large amount of data, while the data volume of the dance database is relatively small. So the image quality is relatively poor.

To solve these problems, this paper proposes a dance action recognition method based on a new Densenet network. Our main contributions are as follows: Firstly, the network model ResNet with good generalization performance is used to extract the deep action features, and its residual module can alleviate the network degradation. Secondly, adopting Exponential Linear Unit (ELU) activation function, Batch Normalization (BN) and Dropout technology to improve the model can reduce gradient disappearance, prevent over-fitting, accelerate convergence and enhance the generalization ability of the model. Finally, the idea of dense network is integrated. The dense connection is added to the multi-layer convolution layer to enhance the richness and effectiveness of the features. The proposed method is tested on public datasets and the self-built dataset. Compared with other existing recognition methods, the experimental results verify the superior performance of the proposed method in practical application.

The arrangement of this paper is as follows: We outline the proposed dance action recognition method in the second section. Section Experiments and Analysis gives the experiments and analysis. Section Conclusions gives a summary of this paper.

## Proposed Dance Action Recognition Method

### Residual Model

**Figure 1:**
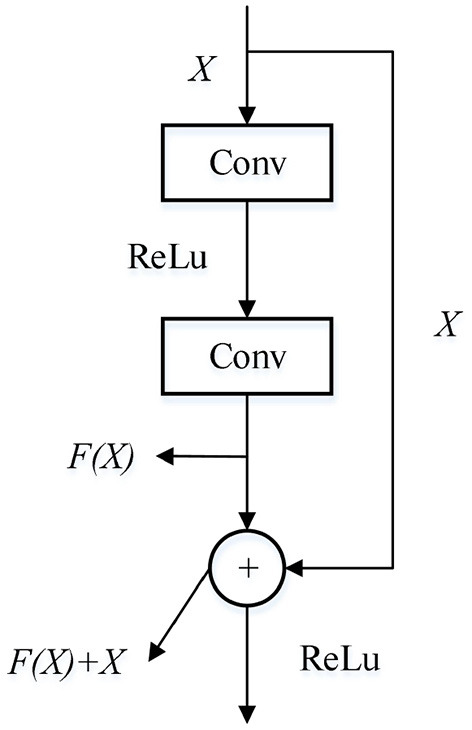
Residual block.

ResNet network is a deep convolutional neural network model (Li et al., 2018b). In this network, when the network is deepened, some problems such as gradient vanishing and gradient explosion will occur, which makes it difficult to train the convolutional neural network. The model performance will also decline (Zhang Y. et al., 2018; Fooladgar and Kasaei, 2020). To mitigate this effect, it can build a Residual block to make skipconnections for different network layers to enhance network performance. Therefore, residual network is widely used in the field of image classification and recognition because of its superior performance. The structure of the residual module is shown in Figure 1.

For a network structure stacked by several layers, when the input data is X, the learning feature is denoted as H(X). It is stipulated that while obtaining H(X), the residual can be obtained through linear transformation and activation function:

(1) $$\begin{array}{r} F\left(X\right)=H\left(X\right)-X \end{array}$$

In this way, the actually learned features are:

(2) $$\begin{array}{r} H_{final}\left(X\right)=F\left(X\right)+X \end{array}$$

Thus, in the extreme case, even if the network layer is a redundant layer, that is, F(X) = 0, the convolution layer realizes the identity mapping. The network performance and feature parameters of the network remain unchanged. In general, F(X) > 0. The network can always learn new features, so as to ensure the gradient transmission in the back propagation and eliminate the problems of network degradation and gradient disappearance.

### Small Convolutional Kernel Residual Network

The convolutional neural network uses the convolutional kernels with different sizes to extract the action features. Then it adopts the full connection layer to fuse the features and extract the deeper feature information. The convolution layer mainly includes two parts: one is the convolution operation in the linear transformation stage; The other part is the activation function operation in the non-linear stage. In here, the convolution kernel is an important part of the convolution layer, which is used to extract the edge, Angle, shape and other features of the image. The activation function mainly introduces non-linearity to enhance the learning ability of the network. With the deepening of network layers, the convolution kernel and training parameters are also increased, so over-fitting is easy to occur in the process of feature extraction (Xiao et al., 2019). The pooling layer in the convolutional neural network can extract representative features for different regions, reduce parameters and improve the computing speed of the network, which can be used as the re-extraction process of output features. Compared with convolution, pooling operation has translational invariant property, and has better robustness to small changes.

It is found that the size of the convolution kernel is larger, the receptive field and the number of required parameters are larger too (Teng et al., 2019). The texture features of action images are usually used for feature recognition. Some different action images have a high similarity in terms of texture features, which mainly relies on small detail features to distinguish. In order to extract subtle features, reduce model parameters as much as possible, significantly improve the performance of the action recognition, and make the model more suitable for real-time application of action recognition, a small convolution kernel residual network is adopted in this paper. It is used to effectively recognize dance action images with 3 × 3 convolution kernel.

**Figure 2:**
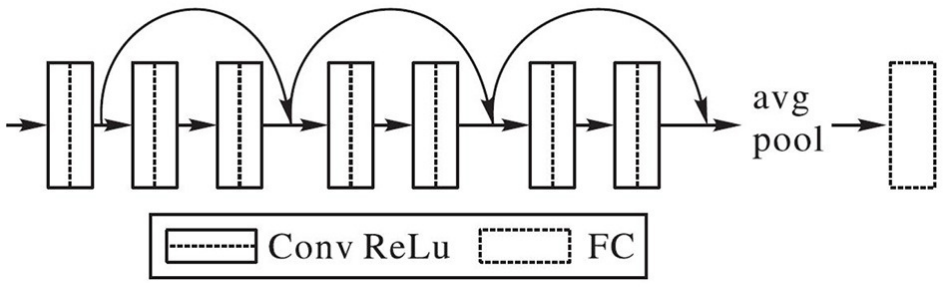
Small convolutional kernel residual network.

In this paper, an improvement is made on the basis of the ResNet network. The convolution kernel of the first layer is set as 3 × 3, and the size of the rest convolution kernel is also set as 3 × 3. Enough convolution layers are added to make up for the impact of the small convolution on receptive field. The classification function of the network adopts Softmax function, and the learning rate is uniformly set at 0.0001. The improved network structure is shown in Figure 2. Based on ResNet-18, it is simplified into eight layers, which greatly reduces model parameters, saves storage space and running time. It is more suitable for the dance action images.

### Activation Function

Activation function mainly solves the linear inseparability problem in neural network, and the superposition of the non-linear activation function after the linear transformation of each layer can make the learning ability stronger and the fitting effect better. The traditional ResNet network adopts the Rectified Linear Unit (ReLU) activation function (Yin and Bi, 2018; Zhang Y. D. et al., 2018). ReLU has the character with simple, Linear and unsaturated. It can effectively alleviate gradient descent and provide sparse expression. The formula of ReLU activation function is as follows:

(3) ReLu(x)={x,x>00,x≤0

As can be seen from Equation (3), when the value of *x* is 1, the gradient will disappear when it is too small. When the value of x is ≤ 0, neuronal apoptosis will occur with the progress of training, resulting in the failure to update the weight.

The ELU activation function combines Sigmod and ReLu with soft saturation on the left side and desaturation on the right side. The right linear part makes the ELU more robust to input variation or noise. The output mean of ELU is close to 0, and the convergence speed is faster, which can solve the problem of neuronal death. The formula of ELU activation function is as follows:

(4) $$ELU(x)=\left\{ \begin{array}{l} x,x>0\\ \alpha (e^{x}-1),x\leq 0 \end{array}\right.$$

**Figure 3:**
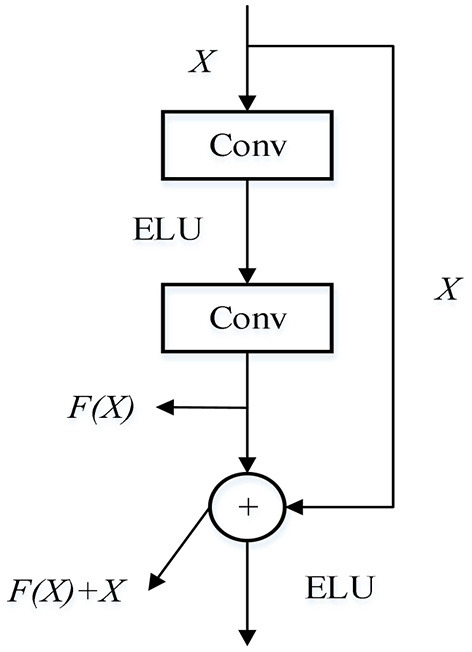
Improved residual block.

The activation function is replaced by ELU to make up for the deficiency of RELU. The unilateral inhibition advantage of RELU is maintained as far as possible, so that the structure of the residual module is improved. The improved residual structure is shown in Figure 3.

### L2 Regularization and Dropout

Batch normalization (BN) makes use of the mean and standard deviation of small batch to continuously adjust the middle output of neural network (Yin et al., 2020), so that the value of the middle output of the whole neural network in each layer is more stable, which can alleviate the over-fitting problem to a certain extent. Secondly, batch normalization can also improve the convergence speed of the model to a certain extent. The Dropout technology was proposed by Hinton (2012). By setting some hidden layer nodes to 0 and ignoring some feature detectors, the performance of the model was improved and the phenomenon of over-fitting was reduced.

That is, in the forward conduction process of the neural network, some neurons are randomly selected to make their activation values temporarily stop working according to a specific probability, so as to increase the generalization ability of the model and prevent the occurrence of over-fitting phenomenon.

### Densely Connected Network

A densely Connected Network (DenseNet) is a neural Network model for optical image processing, which has a powerful feature extraction function (Gao et al., 2020). In the traditional deep network, the extracted features at each layer are equivalent to a non-linear transformation of the input data. Therefore, the complexity of transformation will increase with the network deepening. DenseNet abandons the traditional network connection mode and adopts a relatively densely network connection form. From the perspective of optimal features, feature multiplexing and bypass connection are set directly (Laghari et al., 2019, 2020).

DenseNet makes a network connection directly between one layer and its subsequent layers. In this dense connection mode, the feature graph learned by each layer can be received by subsequent network layers. In other words, each layer in the network accepts the features of all previous layers as input, which is equivalent to that each layer is directly connected to the input layer and the loss layer. Thus, the phenomenon of gradient vanishing can be alleviated, the network structure is more compact, and the extracted features are more abundant. Its output formula is as follows:

(5) $$\begin{array}{r} X_{l}=H_{l}\left(\left[X_{0},X_{1},\cdots \;,X_{l-1}\right]\right) \end{array}$$

Wherein, *X*_*l*_ represents the feature graph mosaic matrix of layer *l*.

**Figure 4:**
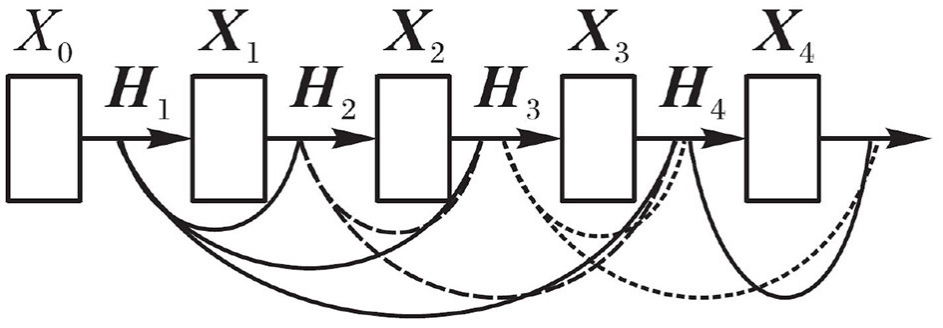
DenseNet connection.

Figure 4 shows the network connection diagram of DenseNet. It can be seen from Figure 4 that the input of any layer in the network is the superposition of all previous layers output. A large number of features are reused, thus enhancing the transmission of features and making the extracted features more abundant. The gradient vanishing is mitigated to a certain extent. In the process of establishing dense connection, when the size of feature graph changes, the layer cannot be directly connected to each other, so the network connection can be established smoothly by changing the size of feature graph with sub-sampling.

**Figure 5:**
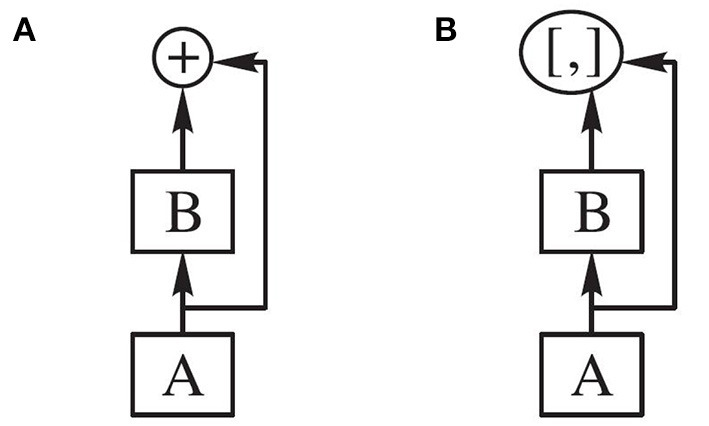
Cross-layer connection. **(A)** Residual connection **(B)** Dense connection.

Although both ResNet and DenseNet use a network connection approach, the residual connection and dense connection are different. The difference is that the Residual connection in ResNet is done by adding between modules. The connection in DenseNet is the connection on the image channel dimension. In Densenet, the growth rate *k* represents the dimension of output feature mapping, where *k* = 12. The cross-layer connection of the network is shown in Figure 5.

## Experiments and Analysis

### Experiment Environment and Evaluation Index

The proposed action recognition neural network is implemented on TensorFlow using Python language. TensorFlow is an open source software library that uses Data Flow Graphs for numerical computation. Equipment parameter: GPU, GTX1080, 16 GB Memory, CPU, Windows10.

In this paper, evaluation indexes: Precision, Recall, F are used to evaluate the proposed algorithm performance (Laghari et al., 2020).

(6) $$\begin{array}{r} Precision\left(i\right)=T\left(i\right)/\left(T\left(i\right)+F\left(i\right)\right) \end{array}$$

(7) $$\begin{array}{r} Recall\left(i\right)=T\left(i\right)/D\left(i\right) \end{array}$$

In Equations (6) and (7), *T*(*i*) represents the number of correctly detected actions of the i-th class. *F*(*i*) represents the number of incorrectly detected actions of the i-th class. *D*(*i*) represents the sample number of i-th class action.

F is the weight average value of precision and recall rate as shown in Equation (8).

(8) F=2·Precison·RecallPrecison+Recall

This section will verify the proposed network in this paper on WISDM, UCI and self-built dance datasets (Laghari et al., 2019). The data is collected by different users performing a range of different activities, such as walking, jogging, climbing stairs, sitting, descending stairs, and standing. 70% of the data set is used for training and 30% for testing. The data sets come from 36, 30, and 30 different users, respectively. The self-built dance action database has 7 dance actions, which are realized by 12 dancers in turn. It includes: up stretch, down stretch, chest cross, fist, move, leg swing, march. The data set is shot in a real environment with light information and partial occlusion. The size of the dance action image is 720 × 480 pixel. We make comparison with other three state-of-the-art action recognition methods including GPRAR (Zhang R. et al., 2018), PGCN-TCA (Xu et al., 2020), MVD (Huynh and Alaghband, 2021).

GPRAR, a graph convolutional network based pose reconstruction and action recognition for human trajectory prediction. The key idea of GPRAR is to generate robust features: human poses and actions, under noisy scenarios. PRAR aims to simultaneously reconstruct human poses and action features from the coherent and structural properties of human skeletons. It is a network of an encoder and two decoders, each of which comprises multiple layers of spatiotemporal graph convolutional networks.

PGCN-TCA: pseudo graph convolutional network with temporal and channel-wise attention. The fixed normalized adjacent matrix is substituted with a learnable matrix. Since frames and input channels that contain outstanding characteristics play significant roles in distinguishing the action from others.

MVD: Dynamic images are extended to the depth domain for action recognition. Multi-view dynamic imaging is proposed for obtaining 3D motion characteristics for action description.

### Accuracy Performance Test

We first make comparison with the activation function ELU and ReLU with/without BN. The classification result is shown in Table 1.

**Table 1:** Results of ELU and ReLU with proposed Densenet.

**Case**	**ELU (%)**	**ReLU (%)**
Without BN	87.1	81.6
With BN	92.3	89.6

From Table 1, we can see that the classification with ELU and BN obtains the best value 92.3% which is superior to other combination. In next experiments, we select ELU+BN.

In order to find the best Dropout value for the dance database, on the basis of the original 8-layer small convolutional kernel ResNet network, only the Dropout layer is added to the average layer, and the image recognition is carried out with this network model. Experiments are carried out on each dance database to calculate the recognition rate, so as to judge the effect of Dropout technology to alleviate over-fitting. In Dropout, p means that each node has p probability to be thrown out, and its value range is (0.1), and the interval is set to 0.1. Table 2 shows the influence of different Dropout values on the recognition rate. As can be seen from Table 2, when the value of Dropout is 0.1, the effect reaches the best on the three databases, which reduces the risk of over-fitting to a certain extent and improves the recognition rate of the model.

**Table 2:** Effects of different Dropout values on different datasets/%.

**Dataset**	**WISDM**	**UCI**	**Self-build**
0.1	98.95	81.96	93.91
0.2	97.43	64.36	88.18
0.3	97.19	76.81	85.96
0.4	97.87	72.81	89.48
0.5	96.19	77.86	83.33
0.6	98.13	68.86	83.21
0.7	97.51	69.31	68.96
0.8	93.67	68.26	83.31
0.9	95.69	63.11	79.86

According to several typical network models such as AlexNet network and GoogleNet network etc., they are used to recognize dance images. The ResNet model is continuously improved. The experiment will be gradually improved for dance recognition, and SK-ResNet, SKResNet+BN, SK-ResNet+BN+ELU, SK-ResNet+BN+ELU+Dropout and the proposed method are recorded successively. The recognition effects with different methods are shown in Table 3. As can be seen from Table 3, for small sample dance databases with different image quality, the recognition rate of the proposed method in this paper is higher than that of other recognition methods.

**Table 3:** Effects of different recognition methods on different datasets/%.

**Dataset**	**WISDM**	**UCI**	**Self-build**
AlexNet	96.37	76.91	80.36
GoogleNet	90.98	56.76	78.69
SK-ResNet	95.15	64.81	77.33
SK-ResNet+BN	96.99	67.12	82.12
SK-ResNet+BN+ELU	98.92	70.12	84.31
SK-ResNet+BN+ELU+Dropout	99.43	82.41	91.15
Proposed	99.98	97.96	97.97

In this experiment, accuracy testing will be conducted on three data sets. The comparative experimental results are shown in Table 4. It can be seen that the accuracy of the proposed recognition model in this paper is higher than other methods on three different data sets. For the WISDM data set, the PGCN-TCA uses PGCN and adds artificially extracted mathematical statistical features in the dense layer, so its accuracy is the best in recent relevant studies. However, the accuracy of proposed models (97.65%) is higher than that of PGCN-TCA without using any artificially features. The effect is more obvious on UCI data set. The accuracy of GPRAR is very low, but the recognition rate of the fused dense network in this paper is much higher than that of other methods. The experimental results also show that the improved dense convolutional structure has better feature extraction ability than other convolutional networks. For the self-build data set, the accuracy of proposed method is 91.55%, which is more than GPRAR (76.54%), PGCN-TCA (79.15%), and MVD (82.37%). Note: the highlighted value is the best result.

**Table 4:** The accuracy results with different methods on different data sets.

**Dataset**	**WISDM (%)**	**UCI (%)**	**Self-build (%)**
GPRAR	89.67	82.51	76.54
PGCN-TCA	94.58	87.18	79.15
MVD	92.37	92.78	82.37
Proposed	97.65	95.89	91.55

### Classification Accuracy Rate

Tables 5–7 show the detailed classification results on WISDM, UCI and self-build datasets. And they are compared with other advanced algorithms. It can be seen that jogging, walking and standing are the easiest to be recognized on WISDM. The accuracy of both the traditional machine learning algorithm and the proposed method in this paper can reach to 90%. Because the two changes are the most distinct from the other actions, they are easily to be recognized. Upstairs and down stairs are hard to recognize. For example, the accuracy of upstairs with GPRAR algorithm can only reach to 71.34%, and the PGCN-TCA can only reach 74.55%, because the two actions are the most easily confused. However, the recognition accuracy of the proposed in this paper exceeds 90%. For UCI and self-build data set, proposed method has more obvious superiority in action classification. It can be concluded that proposed model is much higher than other models in terms of the accuracy and classification accuracy for each action.

**Table 5:** Classification accuracy on WISDM.

**Method**	**GPRAR (%)**	**PGCN-TCA (%)**	**MVD (%)**	**Proposed (%)**
Down stairs	61.23	64.28	72.54	91.25
Jogging	63.47	66.87	76.99	99.74
Sitting	65.96	68.92	78.51	98.52
Standing	69.27	71.26	82.67	96.83
Upstairs	71.34	74.55	85.71	91.65
Walking	76.22	82.38	88.62	98.76

**Table 6:** Classification accuracy on UCI.

**Method**	**GPRAR (%)**	**PGCN-TCA (%)**	**MVD (%)**	**Proposed (%)**
Down stairs	97.79	98.56	96.63	99.89
Lying	99.91	99.24	97.41	100.00
Sitting	89.03	99.35	98.06	100.00
Standing	97.19	87.47	87.31	97.25
Upstairs	99.87	88.63	88.62	99.95
Walking	98.39	99.12	99.67	100.00

**Table 7:** Classification accuracy on self-build dance dataset.

**Method**	**GPRAR (%)**	**PGCN-TCA (%)**	**MVD (%)**	**Proposed (%)**
Up stretch	86.24	87.35	89.57	92.66
Down stretch	85.79	86.81	88.93	91.74
Chest cross	91.25	92.36	94.58	98.63
Fist	92.66	93.76	95.88	98.71
Move	93.78	94.89	96.54	97.25
Leg swing	89.74	90.85	92.76	95.67
March	75.37	76.48	78.69	89.62

### Precision, Recall, F Comparison

In order to observe the performance of proposed model, the precision rate, recall rate and F value are compared on the six actions as shown in Tables 8–10. It can be seen that the precision rate, recall rate and F with proposed model are much higher than the other three structures.

**Table 8:** Precision, recall, and F on WISDM.

**Method**	**Precision (%)**	**Recall (%)**	**F (%)**
GPRAR	93.51	93.59	93.53
PGCN-TCA	94.93	94.73	94.76
MVD	96.57	96.59	96.57
Proposed	97.49	97.45	97.46

**Table 9:** Precision, recall, and F on UCI.

**Method**	**Precision (%)**	**Recall (%)**	**F (%)**
GPRAR	95.44	95.43	95.43
PGCN-TCA	96.14	96.14	96.14
MVD	96.23	97.17	97.16
Proposed	99.13	99.25	99.22

**Table 10:** Precision, recall, and F on self-build dance dataset.

**Method**	**Precision (%)**	**Recall (%)**	**F (%)**
GPRAR	92.37	94.53	93.67
PGCN-TCA	94.61	94.16	94.21
MVD	93.26	96.25	95.54
Proposed	99.47	99.19	99.32

## Conclusions

In this paper, an action image recognition method based on a new dense convolutional neural network is proposed for the dance action. According to the framework of the ResNet network model, the method uses the convolution layer and the pooling layer to extract network features. By using ELU activation function, batch normalization and dropout technology to optimize and improve the model, the gradient disappearance can be alleviated. The over-fitting problem can be prevented, and the convergence can be accelerated. The model generalization ability is stronger. Density connection is added to make the extracted dance action features more abundant and effective. Experimental results on WISDM, UCI and self-built database show that the proposed method can effectively improve the performance of dance action recognition. It is more suitable for practical application of dance action recognition. The method can also be used in other biometric recognition fields, such as palm print recognition, fingerprint recognition, face recognition and so on. The next step will focus on the research and improvement of the new network model in order to obtain better recognition effect.

## Data Availability Statement

The original contributions presented in the study are included in the article/supplementary material, further inquiries can be directed to the corresponding author/s.

## Author Contributions

XY and YL: drafting and refining the manuscript. YS and CZ: critical reading of the manuscript. All of the authors have read and approved the manuscript.

## Conflict of Interest

The authors declare that the research was conducted in the absence of any commercial or financial relationships that could be construed as a potential conflict of interest.
